# Transcriptional signatures associated with environmental and developmental variation in tobacco aroma-related pathways across cultivars and regions

**DOI:** 10.3389/fpls.2026.1854430

**Published:** 2026-10-21

**Authors:** Xiaoxu Li, Yilin Wen, Xinxi He, Jiashuo Yang, Guoping Wang, Yu Peng, Zhiyuan Li, Yu Zhao, Dousheng Wu, Xiangli Xu, Wanfeng Liu, Yangyang Li, Pingjun Huang, Junping Gao, Yirong Wang, Long Chen, Xiaonian Yang, Wenxuan Pu

**Affiliations:** 1 Technology Center of China Tobacco Hunan Industrial Co. Ltd., CNTC, Changsha, China; 2 Beijing Life Science Academy, Beijing, China; 3 College of Biology, Hunan University, Changsha, China; 4 Hunan Tobacco Research Institute, Changsha, China; 5 Yuxi Zhongyan Tobacco Seed Co., Ltd, Yuxi, Yunnan, China

**Keywords:** environmental adaptation, gene expression variability, nicotiana tabacum, secondary metabolism, tobacco aroma, transcriptome

## Abstract

**Background:**

Tobacco aroma is a complex quality trait shaped by interactions among genotype, developmental progression, and regional environment, yet the transcriptional programs linking ecological context to aroma-related metabolism remain incompletely understood.

**Methods:**

We profiled the transcriptomes of ten *Nicotiana tabacum* L. cultivars grown in three representative regions and sampled at three developmental stages. Normalized RNA-seq expression, principal component analysis, differential expression analysis, soft clustering, pathway enrichment, gene-expression variability analysis, and protein–protein interaction network analysis were performed.

**Results:**

Growing region was the major source of transcriptomic variation, followed by developmental stage, whereas cultivar effects were comparatively smaller. Leaf maturation from S1 to S3 was associated with repression of photosynthesis and carbon-assimilation pathways and induction of stress responses, redox regulation, cell-wall and membrane remodeling, amino acid metabolism, and secondary metabolism. Aroma-associated processes, including carotenoid-, phenylpropanoid-, and redox-related pathways, showed regiondependent expression patterns. Strong-aroma cultivars exhibited greater transcriptional variability across regions than light-aroma cultivars, suggesting stronger environmental responsiveness of their regulatory networks. Network analysis further highlighted candidate hub genes, including the heat shockrelated protein A0A1S4A548 and peroxiredoxin Q A0A1S4B900, in temperature- and oxidative-stress-related modules.

**Conclusion:**

These findings provide a transcriptome-level framework for understanding how environmental context and developmental maturation are associated with aroma-related metabolic reprogramming in tobacco and identify candidate genes for future functional validation.

## Introduction

Tobacco (*Nicotiana tabacum* L.), a member of the *Solanaceae* family, is an important non-food economic crop worldwide. In addition to being well known for the production of alkaloids such as nicotine, whose biosynthesis is tightly regulated during plant growth and agricultural treatment, tobacco leaves accumulate a wide range of aroma-related metabolites that contribute to leaf quality, sensory characteristics, and commercial value (Tong et al., 2016; Qin et al., 2020). Tobacco aroma is not determined by a single compound or pathway, but rather represents a complex quantitative trait shaped by genetic background, developmental progression, environmental conditions, and post-harvest processing.

The formation of tobacco aroma depends on the coordinated synthesis, transformation, and accumulation of multiple biochemical components, including carbohydrates, pigments, polyphenols, carotenoid-derived volatiles, phenylpropanoid-related compounds, terpenoids, and other volatile organic compounds in leaf tissues (Banozic et al., 2020; Popova et al., 2019). Carbohydrates provide important precursors and participate in Maillard-type reactions and other chemical processes associated with flavor development, whereas carotenoids and phenylpropanoids are major sources of aroma-related volatile compounds (Banozic et al., 2020; Popova et al., 2019; Dong and Lin, 2021). Previous metabolomic and proteomic studies have shown that the abundance and distribution of aroma precursors vary among tobacco tissues, cultivars, and stalk positions, and developmental stages, indicating that aroma formation is closely linked to both intrinsic genetic programs and developmental regulation (Chang et al., 2020; Liu et al., 2022a; Zhang et al., 2018). Environmental factors, including soil properties, climate, cultivation region, and microbial communities, further modulate tobacco quality by influencing carbon allocation, stress responses, and secondary metabolism (Zhang et al., 2022; Yan et al., 2020). Despite recognition of these complex interactions, the transcriptional regulatory programs that coordinate environmental signals with aroma-related metabolism across different cultivars and growing regions remain largely unexplored.

Recent advances in genomics, transcriptomics, and bioinformatics have provided powerful tools for dissecting the regulatory networks underlying complex plant traits (Zhou et al., 2025; Jia et al., 2025; Zheng et al., 2026; Li et al., 2025). RNA sequencing enables systematic profiling of gene expression across developmental stages, tissues, cultivars, and environmental conditions, thereby facilitating the identification of candidate genes, pathways, and regulatory modules associated with metabolic and agronomic traits (Stark et al., 2019). In tobacco, transcriptomic and multi-omics analysis have been applied to study leaf development, pollen and pollen tube growth, flower color variation, cytoplasmic male sterility, nicotine biosynthesis, and responses to abiotic or biotic stresses (Chang et al., 2020; Conze et al., 2017; Jiao et al., 2020; Liu et al., 2020; Qin et al., 2020; Khan et al., 2023; Luo et al., 2022; Li et al., 2018). Studies related to tobacco aroma have primarily focused on the identification of aroma compounds and precursors, environmental effects on aroma-associated metabolites, and strategies for improving flavor quality through microbial fermentation or enzymatic treatment (Chida et al., 2004; Liu et al., 2022a; Yan et al., 2020; Rodriguez-Bustamante et al., 2005; Gong et al., 2023; Ning et al., 2023). However, compared with metabolite profiling and post-harvest improvement strategies, the transcriptional regulatory programs that connect environmental variation with aroma-related metabolic pathways across cultivars and growing regions remain insufficiently characterized.

In this study, we aimed to characterize transcriptomic signatures associated with developmental progression and regional environmental variation across multiple tobacco cultivars. By integrating large-scale RNA-seq profiling of ten cultivars grown in three geographically distinct regions and sampled at defined developmental stages, we sought to identify candidate genes, biological pathways, and regulatory modules potentially involved in environmentally responsive aroma-related metabolic processes. Although direct quantification of aroma compounds was beyond the scope of this study, the large-scale comparative transcriptomic approach enables the prioritization of candidate hub genes and regulatory modules that are most likely to govern environment-responsive aroma formation, providing a roadmap for targeted functional validation and cultivation optimization.

## Materials and methods

### Plant materials and sample collection

Ten tobacco cultivars of *Nicotiana tabacum* L. were used in this study, including K326, Yueyan96, Hongda, Nanjiang3, Zhongyan103, Zhongyan104, Yunyan2, Yunyan87, Yunyan85, and G30. Plant materials were collected from three representative tobacco-growing regions: Guiyang County, Chenzhou, Hunan Province (CZ); Longshan County, Xiangxi, Hunan Province (LS); and Jiangchuan District, Yuxi, Yunnan Province (YX). Samples were collected at three developmental stages where available: the central flowering period (S1), the mature stage of the peduncle (S2), and the middle leaf maturity stage (S3).

For each sample, leaf mesophyll tissues were rapidly collected, immediately frozen in liquid nitrogen, and stored at −80 °C until RNA extraction. To reduce variation caused by small-scale environmental heterogeneity within the field, mesophyll tissues from three individual plants grown under the same region-cultivar-stage condition were pooled to generate one biological replicate for RNA-seq library construction. In total, 380 biological replicates/RNA-seq libraries were included in this study. The sampling design covered all three regions and all three developmental stages, but the number of biological replicates differed among some region-cultivar-stage combinations. Most combinations contained four biological replicates, whereas the YX region included additional biological replicates at the S3 stage for all ten cultivars. Specifically, the total sample numbers were 98 in CZ, 121 in LS, and 161 in YX. Detailed information on the sampling regions, cultivar names, developmental stages, replicate numbers, and sample IDs is provided in **Supplementary Table 1**.

### RNA collection, library construction and sequencing

Total RNA was extracted from all tobacco leaf samples using TRIzol reagent according to manufacturer’s instructions (Invitrogen). RNA quantity and quality were assessed before library construction, and only RNA samples with sufficient integrity and purity were used for subsequent sequencing. For each sample, 1 μg of total RNA was used as input material for RNA-Seq library preparation. Sequencing libraries were generated using the NEBNext^®^ Ultra™ RNA Library Prep Kit for Illumina^®^ (NEB) following the manufacturer’s recommendations, and index codes were added to attribute sequences to each sample. The final library was sequenced as 150 bp paired-end reads on Illumina NovaSeq 6000 platform.

### RNA-seq data processing and differential expression analysis

The clean reads were mapped to the tobacco reference genome *Nicotiana tabacum*2017 (https://solgenomics.net/) using HISAT2 (Kim et al., 2019). Uniquely mapped reads were summarised to annotated genes using featureCounts (Liao et al., 2014). The raw gene expression matrix was imported into the R environment for further processing and analysis. Genes with very low read abundance were filtered out before statistical analysis. Specifically, genes with fewer than 50 total reads across all libraries included in the corresponding analysis were removed. After filtering, approximately 25,000 expressed genes were retained for downstream comparisons. For exploratory and visualization analysis, including principal component analysis (PCA), hierarchical clustering, and expression pattern comparisons, gene expression values were normalized as fragments per kilobase of transcript per million mapped fragments (FPKM), which accounts for both sequencing depth and gene length. FPKM values were log2-transformed as log2(FPKM + 1) before PCA and clustering analysis.

Differential expression analysis was performed using the DESeq2 package (Love et al., 2014). Differentially expressed genes (DEGs) were identified based on two criteria: Benjamini–Hochberg adjusted P value, also referred to as false discovery rate (FDR), <0.01 and absolute value of log2(Fold Change) >1. Hierarchical clustering was performed using Pearson correlations calculated from normalized expression values. DEGs were subsequently subjected to KEGG pathway enrichment analysis using the clusterProfiler package (Yu et al., 2012).

### Dynamic expression pattern analysis

To characterize dynamic gene expression patterns associated with developmental progression and regional variation, soft clustering analysis was performed using the Mfuzz package (Kumar and M, 2007). Unlike hard clustering methods, which assign each gene to a single cluster, soft clustering allows genes to have graded membership values across clusters and is therefore more suitable for noisy or continuous transcriptomic data. To investigate developmental stage-associated expression trends, genes showing differential expression across developmental stages were analyzed across S1, S2, and S3. The Mfuzz parameters were set as follows: minimum standard deviation = 0.3; membership score threshold = 0.5, and optimal cluster number c = 6. Genes assigned to each cluster were then used for Gene Ontology (GO) and KEGG pathway enrichment analysis to interpret the biological processes and pathways represented by different temporal expression patterns.

Similarly, to explore the influence of regional environmental variation on gene expression patterns, Mfuzz clustering was applied to region-associated expression data from CZ, LS, and YX. Genes with similar region-dependent expression profiles were grouped into clusters, followed by GO and KEGG enrichment analysis for each cluster. These analyses were used to identify biological processes and metabolic pathways potentially associated with developmental progression and regional environmental responses.

### Protein-protein interaction network construction

To further explore candidate regulatory genes within aroma-related biological processes, protein–protein interaction (PPI) networks were constructed for selected functional modules. Based on the GO enrichment results, aroma-related pathways or biological processes were selected for downstream network analysis. Only pathways or modules containing at least 20 genes were retained to ensure sufficient network complexity. The corresponding genes were submitted to the STRING database to infer protein–protein associations, with the minimum required interaction confidence score set to 0.4 (Szklarczyk et al., 2023). The resulting interaction networks were imported into Cytoscape software version 3.9.1 for visualization and further refinement (Shannon et al., 2003). Candidate hub genes were identified based on their network connectivity and biological relevance to stress responses, redox regulation, hormone signaling, and aroma-related metabolic pathways.

## Results

### Profiling scheme in tobacco strains, stages, and regions

To investigate the transcriptomic basis of tobacco aroma variation, we analyzed ten cultivars of *Nicotiana tabacum* collected from three representative tobacco-growing regions in China: Chenzhou (CZ), Longshan (LS), and Yuxi (YX) (**Figure 1A**). Samples were collected at three developmental stages, including the central flowering period (S1), the mature stage of the peduncle (S2), and the middle leaf maturity stage (S3). In total, 380 RNA-seq libraries were generated, representing multiple cultivars, developmental stages, and growing regions (**Supplementary Table 1**).

**Figure 1:**
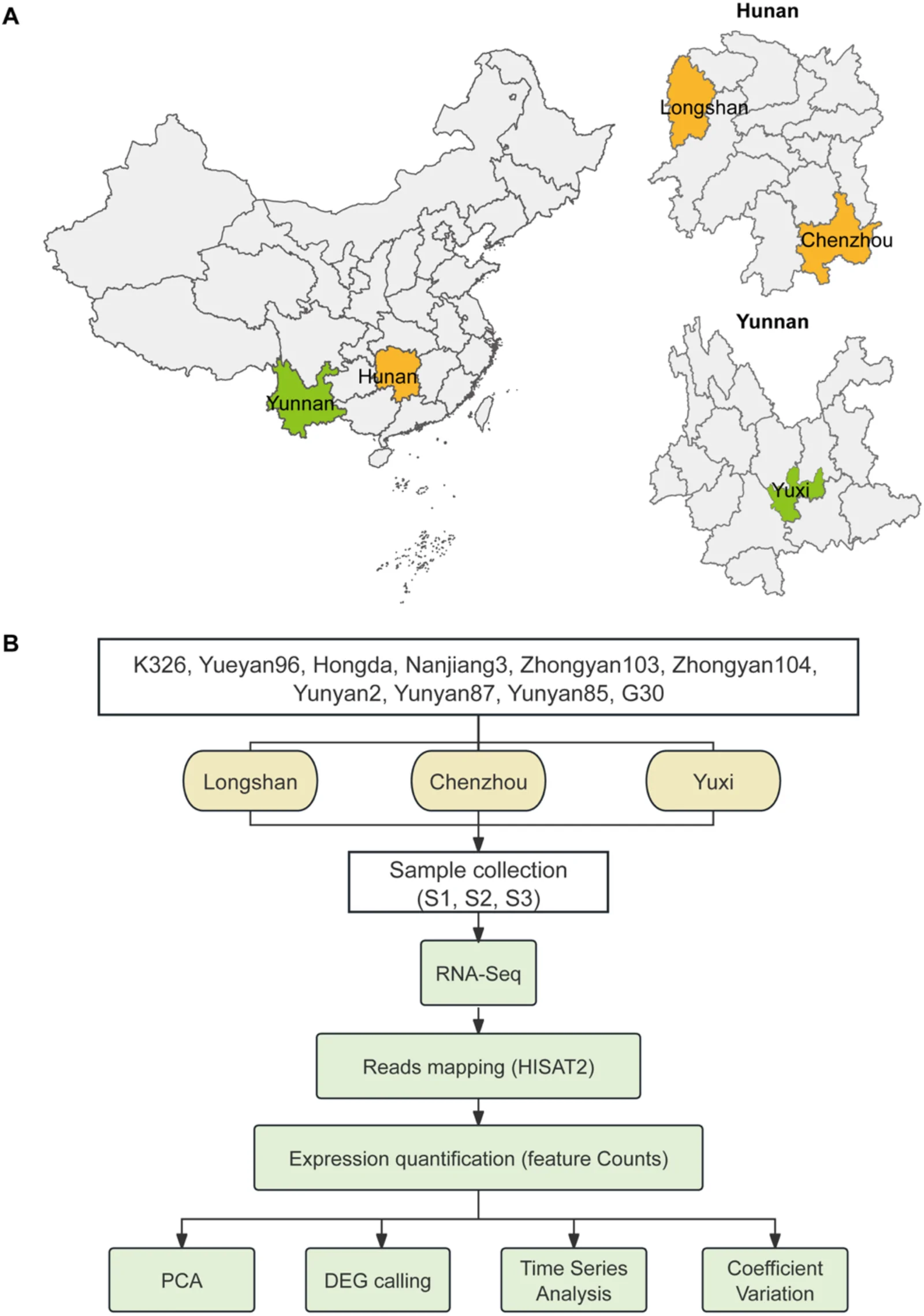
Study design and transcriptomic analysis strategy. **(A)** Locations of the three tobacco-growing regions used for sample collection: Chenzhou (CZ), Hunan Province, Longshan (LS), Hunan Province, and Yuxi (YX), Yunnan Province. Ten Nicotiana tabacum cultivars were included in the study. **(B)** Schematic overview of the RNA-seq workflow and downstream analysis. Samples were collected at three developmental stages, including the central flowering period (S1), mature stage of the peduncle (S2), and middle leaf maturity stage (S3). Transcriptomic data were analyzed using PCA, differential expression analysis, Mfuzz clustering, functional enrichment analysis, and CV analysis.

The overall study design and analytical workflow are summarized in **Figure 1B**. Following RNA sequencing and expression quantification, normalized gene expression values (FPKM) were used to characterize global transcriptomic variation by principal component analysis (PCA). To identify genes associated with developmental remodeling, differential expression analysis was performed between S1 and S3 samples from the same cultivar and region, representing the largest developmental contrast. In parallel, Mfuzz soft clustering was applied to all three developmental stages (S1–S3) to identify dynamic expression patterns during leaf development, whereas the coefficient of variation (CV), defined as the ratio of the standard deviation (σ) to the mean (μ) of gene expression levels, was used to evaluate transcriptomic variability among cultivars. Together, these complementary analysis enabled systematic characterization of transcriptome variation associated with developmental progression, regional environmental differences, and cultivar diversity.

### Growing region is the dominant source of transcriptomic variation

To evaluate the major sources of transcriptomic variation in tobacco leaves, we first examined global gene expression patterns across the 380 RNA-seq libraries. After expression quantification, 20,466–23,036 expressed genes were detected per sample, and the overall expression distributions were broadly comparable across cultivars, developmental stages, and regions, indicating that the dataset was suitable for comparative transcriptomic analysis (**Figure 2A**).

**Figure 2:**
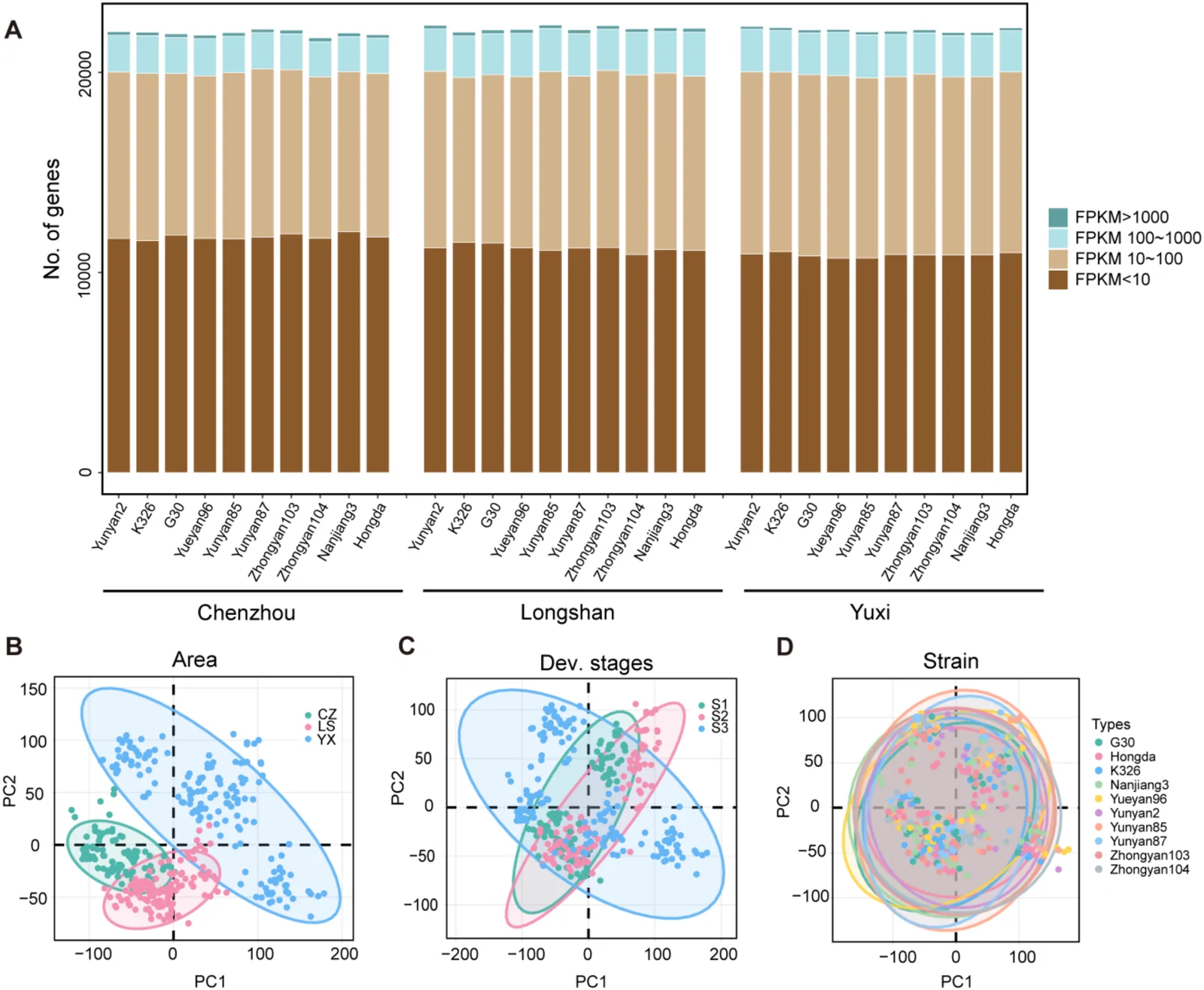
Global gene expression profiles and major sources of transcriptomic variation. **(A)** Distribution of expressed genes across the ten tobacco cultivars grown in Chenzhou, Longshan, and Yuxi. Genes were classified into four expression-level groups according to FPKM values: <10, 10–100, 100–1,000, and >1,000. **(B–D)** Principal component analysis (PCA) of tobacco leaf transcriptomes based on normalized FPKM values. Samples are colored according to growing region in **(B)**, developmental stage in **(C)**, and cultivar identity in **(D)**. Ellipses represent the 95% confidence intervals for each group, illustrating the relative contribution of region, developmental stage, and cultivar to global transcriptomic variation.

PCA based on normalized FPKM values revealed a strong regional effect on the tobacco leaf transcriptome. Samples from the three growing regions, CZ, LS, and YX, formed clearly separated clusters, suggesting that geographical origin and associated environmental conditions represented the major source of global expression variation in this dataset (**Figure 2B**). Developmental stage also contributed substantially to transcriptomic differences. Samples from S1 and S2 were relatively close to each other, whereas S3 samples were separated from the earlier stages, indicating a major transcriptional transition during leaf maturation (**Figure 2C**). By contrast, cultivar-associated separation was much less pronounced, as samples from different cultivars largely overlapped in the PCA space(**Figure 2D**).

Further stratified PCA analysis supported these observations. When samples were examined within the same developmental stage, samples from the same region tended to cluster together, although the separation was less clear at S3, suggesting that mature leaves may show more complex transcriptomic variation (**Supplementary Figure S1A**). Within individual regions, early-stage samples (S1 and S2) were generally separated from mature-stage samples (S3), particularly in LS and YX, further supporting the developmental shift observed in the full dataset (**Supplementary Figure S1B**). In contrast, cultivar-specific clustering remained weak within regions, especially in LS and CZ, indicating that regional and developmental effects were stronger than cultivar effects under the present sampling design (**Supplementary Figure S1C**).

Together, these results indicate that growing region was the dominant factor shaping tobacco leaf transcriptomic variation, followed by developmental stage, whereas cultivar identity contributed comparatively less to global expression differences. These findings suggest that environmental context and developmental progression strongly influence gene expression programs in tobacco leaves and provide a basis for subsequent analysis of aroma-related metabolic pathways and regulatory modules.

### Developmental-stage effects on aroma-related transcriptional programs

Developmental progression is closely associated with metabolic remodeling in tobacco leaves and contributes to changes in leaf quality and aroma-related precursor accumulation (Liu et al., 2022b; Zhang et al., 2018). To investigate developmental-stage effects on gene expression dynamics, Mfuzz soft clustering was performed using gene expression profiles across S1, S2, and S3 within each cultivar and region. Six major expression clusters were identified, representing distinct temporal patterns during leaf development (**Figure 3A**). The complete clustering patterns across cultivars and regions are shown in **Supplementary Figures S2**–**S4**, and the corresponding gene information for each cluster is provided in **Supplementary Table 2**.

**Figure 3:**
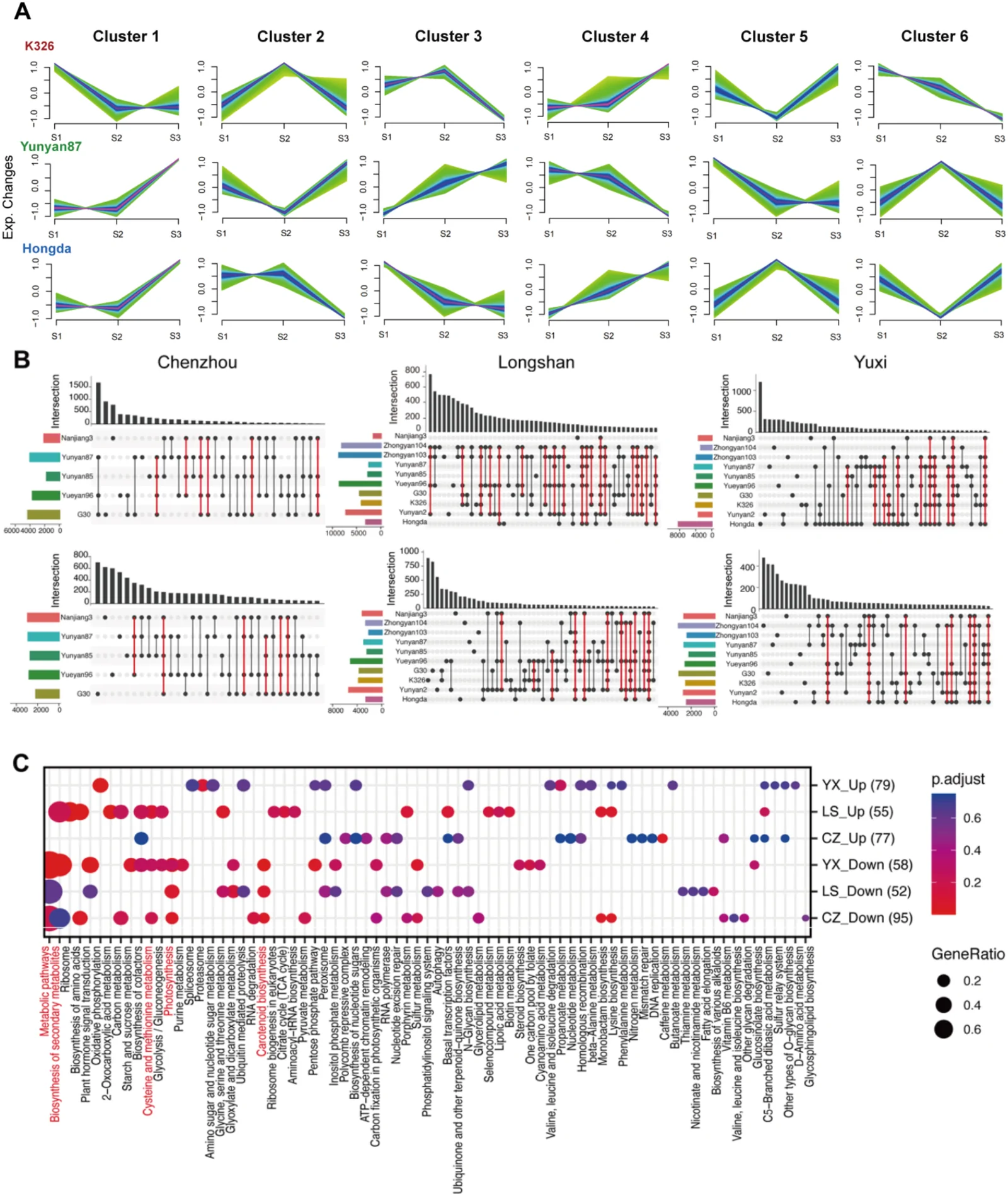
Developmental-stage-dependent transcriptional dynamics and pathway enrichment across growing regions. **(A)** Mfuzz soft clustering of gene expression profiles across the three developmental stages, S1, S2, and S3. Representative results are shown for K326, Yunyan87, and Hongda from the Longshan region. Six major clusters were identified, reflecting distinct temporal expression trajectories, including progressively increased, progressively decreased, and non-monotonic patterns during leaf development. **(B)** UpSet analysis of genes showing gradual expression changes during development. The overlap of upregulated and downregulated developmental gene sets across cultivars is shown separately for Chenzhou, Longshan, and Yuxi. Gene sets shared by more than four cultivars were considered conserved developmental signals within each region and were used for downstream enrichment analysis. **(C)** KEGG pathway enrichment analysis of shared developmental gene sets. Upregulated and downregulated genes from each region were analyzed separately. The x-axis shows enriched KEGG pathways, and the y-axis indicates region-specific gene sets. Dot size represents the gene ratio, while dot color indicates the adjusted P value. The results highlight both shared and region-dependent transcriptional remodeling during tobacco leaf development.

For example, in the strong-aroma cultivar K326 from the Longshan region, cluster 6 showed a gradual decrease from S1 to S3, whereas cluster 4 showed a progressive increase. In contrast, the light-aroma cultivar Hongda displayed different temporal patterns in clusters 1, 2, 5, and 6 compared with K326, suggesting cultivar-dependent developmental transcriptional programs (**Figure 3A**; **Supplementary Figures S2**–**S4**). Genes showing continuous upregulation or downregulation across S1–S3 were further examined, as these genes may reflect maturation-associated transcriptional remodeling relevant to aroma-related metabolism.

To determine whether developmental-stage-associated genes were shared across cultivars and regions, UpSet analysis was performed for genes showing gradual expression changes during development. The results showed that most gene sets were shared by only two or three cultivars within each region, whereas genes showing gradual decreases during maturation were largely cultivar-specific (**Figure 3B**). To focus on more conserved developmental signals, genes shared by more than four cultivars within the same region were selected for downstream functional enrichment analysis.

Functional enrichment analysis revealed both common and region-dependent features of developmental transcriptional remodeling. In CZ, 698 upregulated and 856 downregulated genes were identified among the shared developmental gene sets. In LS, 244 upregulated and 396 downregulated genes were identified, whereas in YX, 486 upregulated and 359 downregulated genes were detected. In all three regions, downregulated genes were mainly enriched in photosynthesis-, chloroplast-, and thylakoid membrane-related processes, indicating a general reduction in photosynthetic activity during leaf maturation. In contrast, upregulated genes showed more region-specific enrichment patterns. In LS, upregulated genes were enriched in ribosome-related pathways and secondary metabolism, whereas in YX, they were mainly associated with mitochondrial functions (**Figure 3C**). Similar functional trends were observed in GO enrichment analysis (**Supplementary Figure S5**).

Overall, developmental progression was accompanied by broad transcriptional reprogramming in tobacco leaves, characterized by downregulation of photosynthesis- and chloroplast-related pathways and a shift toward metabolism-, ribosome-, and organelle-associated processes. The magnitude and functional direction of these changes varied among growing regions, suggesting that developmental programs interact with regional environmental context to shape aroma-related transcriptional variation.

### Differential expression analysis reveals maturation-associated metabolic remodeling

The PCA results showed clear separation between S3 samples and those from the earlier developmental stages S1 and S2, indicating substantial transcriptomic remodeling during late leaf maturation (**Figure 2C**; **Supplementary Figure S1**). To identify genes associated with this developmental transition, we performed pairwise differential expression analysis between S3 and S1 samples within each cultivar and region. Across comparisons, 877 to 4,870 DEGs were identified, with upregulated genes generally representing a larger proportion than downregulated genes (**Supplementary Figure S6**). These results indicate that the transition from the early developmental stage to the mature leaf stage involves extensive transcriptional reprogramming.

To investigate the biological processes associated with these developmental changes, KEGG enrichment analysis was performed for the DEGs from each comparison. The top enriched pathways are summarized in **Figure 4**. Overall, upregulated genes were mainly enriched in pathways related to general metabolism, cell wall and membrane biosynthesis, stress responses, amino acid metabolism, and secondary metabolite biosynthesis. These pathways included glycerolipid metabolism, glycosphingolipid biosynthesis, nucleotide sugar biosynthesis, galactose metabolism, sphingolipid metabolism, arginine and proline metabolism, nitrogen metabolism, glutathione metabolism, and biosynthesis of secondary metabolites. These enrichment patterns suggest that leaf maturation is accompanied by enhanced metabolic activity, structural remodeling, and stress-related responses.

**Figure 4:**
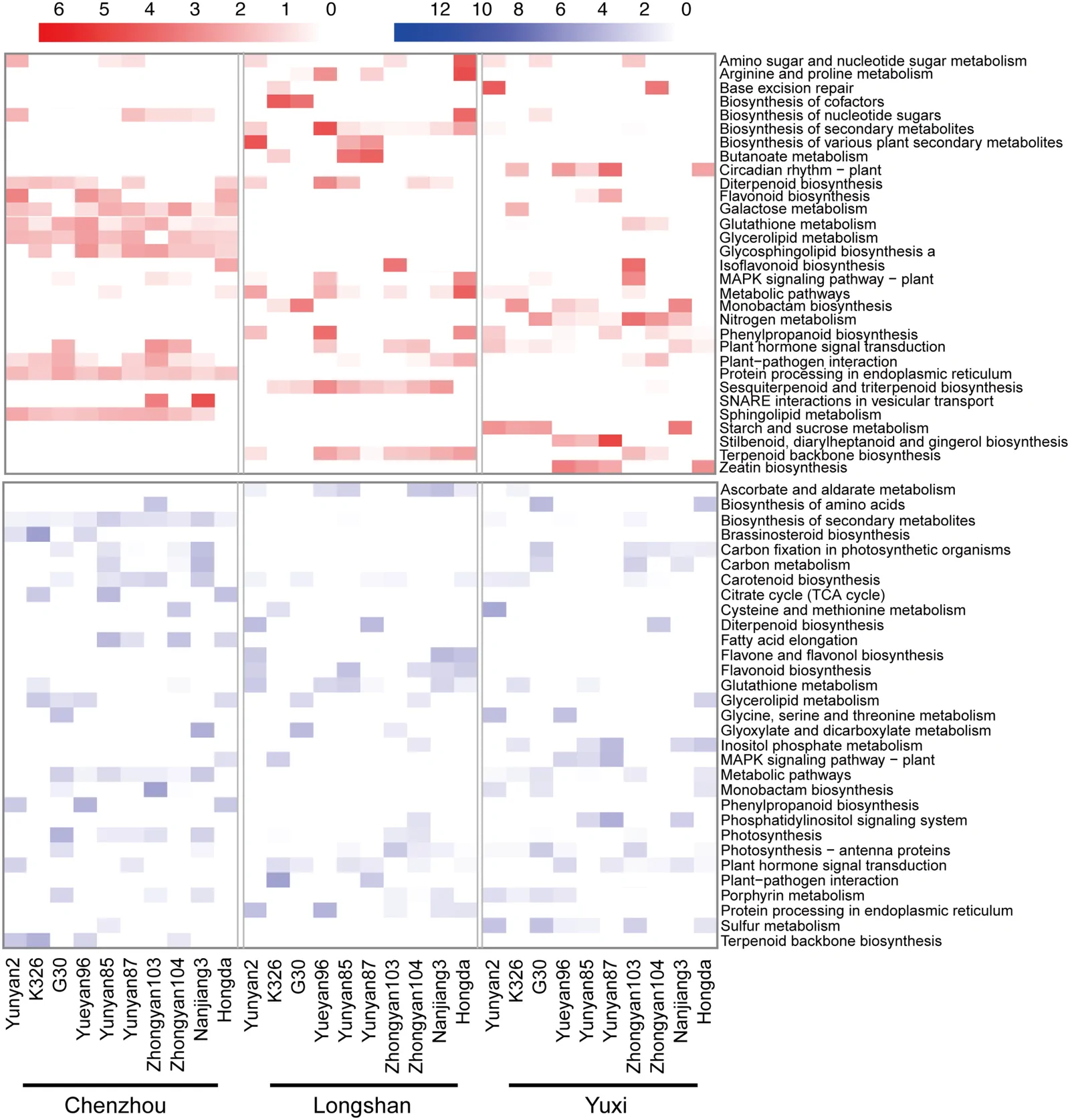
Functional enrichment analysis of DEGs across cultivars and growing regions. KEGG enrichment heatmap of differentially expressed genes identified by comparing S3 with S1 samples within each cultivar and region. Columns represent cultivars grouped by Chenzhou, Longshan, and Yuxi, and rows represent enriched KEGG pathways. Red indicates pathways enriched among upregulated DEGs, while blue indicates pathways enriched among downregulated DEGs. Color intensity represents the enrichment score, with darker colors indicating stronger pathway enrichment.

Conversely, downregulated genes were predominantly enriched in photosynthesis- and carbon metabolism-related pathways. These included photosynthesis, photosynthesis antenna proteins, carbon fixation in photosynthetic organisms, carotenoid biosynthesis, and broader carbon metabolism pathways (**Figure 4**). The reduced expression of genes associated with photosynthetic and chloroplast-related functions is consistent with the developmental transition from active growth toward mature leaf physiology. Because carotenoid metabolism and carbon allocation are closely connected to the formation of aroma-related precursors, these transcriptional changes may be relevant to the metabolic remodeling associated with tobacco leaf maturation.

Notably, comparisons from the same growing region showed broadly similar enrichment patterns, whereas the strength and composition of enriched pathways differed among regions. This suggests that developmental-stage-associated transcriptional remodeling is influenced by regional environmental context. Together, these results indicate that tobacco leaf maturation is characterized by coordinated upregulation of metabolic, structural, and stress-responsive pathways, alongside downregulation of photosynthesis- and carbon fixation-related pathways.

### Transcriptomic variability differs among aroma-type cultivars and regions

The preceding analysis indicated that growing region and developmental stage were major contributors to transcriptomic variation in tobacco leaves. To further assess whether cultivars with different aroma characteristics exhibited distinct levels of transcriptional variability, we calculated the CV of gene expression across samples for each cultivar. The CV was defined as the ratio of the standard deviation to the mean expression level and was used here as an indicator of transcriptomic variability across environmental and developmental conditions.

The distribution of gene expression CVs differed markedly among cultivars (**Figure 5A**). Notably, K326 and Yueyan96, are generally characterized as strong-aroma cultivars, showed higher median CV values, whereas Hongda, a light-aroma cultivar, showed the lowest median CV. These results suggest that strong-aroma cultivars may exhibit more variable or environmentally responsive transcriptional programs, while the light-aroma cultivar Hongda may maintain a relatively stable expression profile across conditions. To further compare cultivars representing different aroma types, we selected K326, Yunyan87, and Hongda as representative strong-, medium-, and light-aroma cultivars, respectively. Consistent with the overall cultivar-level analysis, these three representatives showed significant differences in gene expression CV, with Hongda displaying lower transcriptomic variability than the other two cultivars (**Figure 5B**). These representative cultivars were therefore used for subsequent comparative analysis.

**Figure 5:**
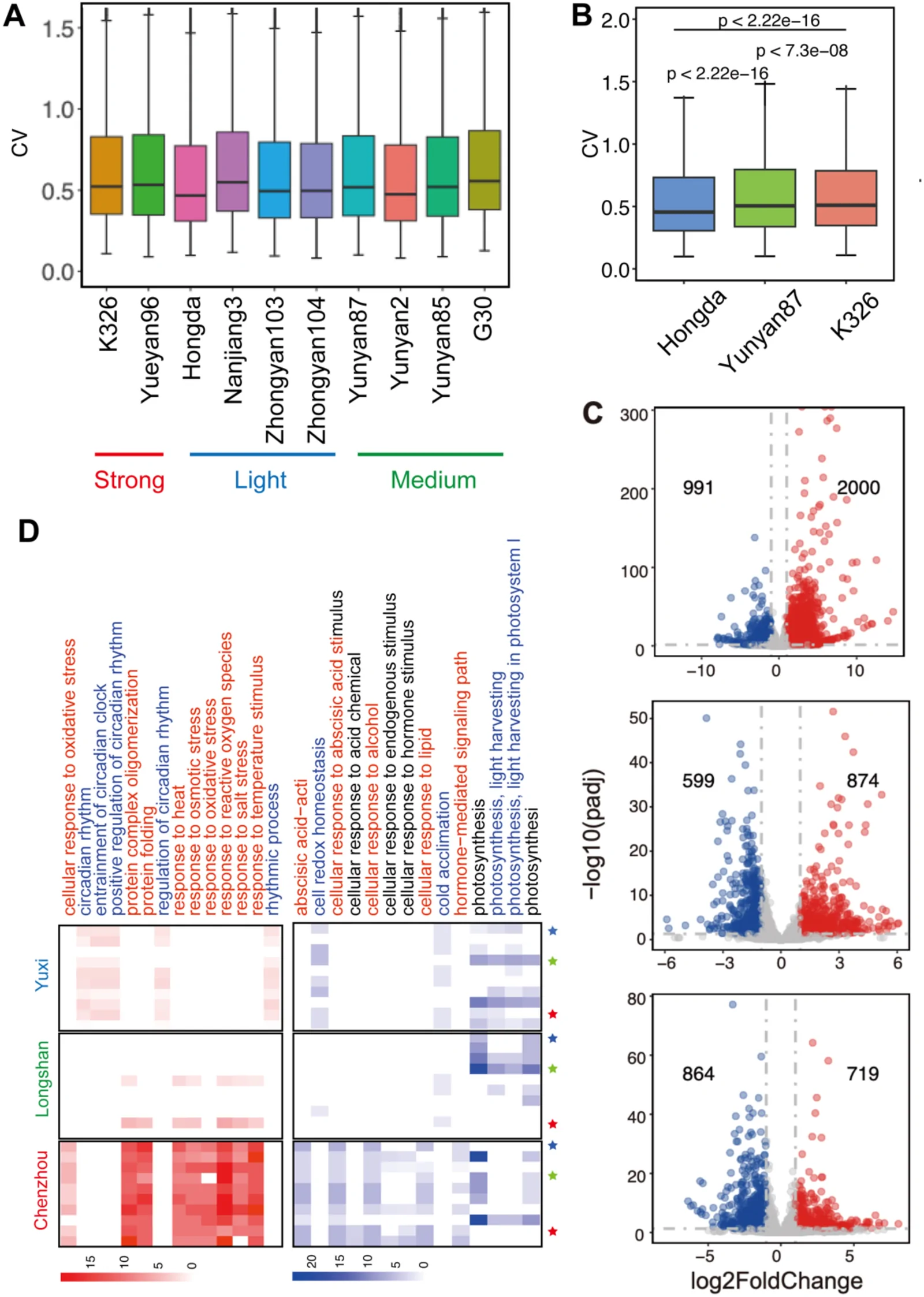
Transcriptomic variability and functional enrichment in representative aroma-type cultivars. **(A)** Distribution of gene expression coefficient of variation (CV) across all samples for each tobacco cultivar. Cultivars are grouped according to their reported aroma characteristics: strong-aroma, light-aroma, and medium-aroma types. **(B)** Comparison of gene expression CVs among three representative cultivars: Hongda, light-aroma; Yunyan87, medium-aroma; and K326, strong-aroma. P values indicate statistical significance between groups. **(C)** Volcano plots showing differentially expressed genes (DEGs) between S3 and S1 in the three representative cultivar-region combinations. From top to bottom: K326 in Chenzhou, Yunyan87 in Longshan, and Hongda in Yuxi. Red and blue points indicate upregulated and downregulated DEGs, respectively, and the numbers indicate the corresponding DEG counts. **(D)** Functional enrichment heatmap of DEGs from the three representative comparisons. Enriched terms are grouped by cultivar-region comparison, and red and blue indicate enrichment among upregulated and downregulated DEGs, respectively. Color intensity represents the enrichment score. The results highlight cultivar- and region-dependent differences in developmental transcriptional remodeling, including stress response, redox regulation, protein homeostasis, and hormone-related pathways.

We next compared developmental transcriptional changes between S3 and S1 in the three representative cultivars grown in representative regions. In K326 from Chenzhou, 2,000 genes were upregulated and 991 genes were downregulated at S3 compared with S1. In Yunyan87 from Longshan, 874 genes were upregulated and 599 genes were downregulated, whereas in Hongda from Yuxi, 719 genes were upregulated and 864 genes were downregulated (**Figure 5C**). These results indicate that the magnitude and direction of developmental transcriptional remodeling differed among aroma-type cultivars and regional contexts.

Functional enrichment analysis further revealed distinct biological features among the three representative comparisons. In K326 from Chenzhou, upregulated genes were significantly enriched in terms related to protein complex oligomerization, protein folding, response to heat, osmotic stress, oxidative stress, reactive oxygen species, and temperature stimulus (**Figure 5D**). K326 also showed the largest number of significantly changed functional terms, consistent with its higher transcriptomic variability. By contrast, Hongda from Yuxi did not show strong enrichment of these stress- and protein homeostasis-related terms. Instead, the significantly changed terms in Hongda were mainly associated with circadian rhythm and redox homeostasis. Together, these results suggest that cultivars with different aroma characteristics may differ in their transcriptional plasticity and in the biological pathways through which they respond to developmental and regional environmental cues.

### Candidate hub genes in aroma-related environmental response modules

To further prioritize candidate genes within biological processes potentially associated with aroma-related environmental responses, we constructed protein–protein interaction (PPI) networks for selected enriched functional modules. Because the preceding analysis highlighted stress response, redox regulation, and hormone signaling as major components of developmental and regional transcriptomic variation, we focused on three representative modules: response to temperature stimulus, response to oxidative stress, and hormone-mediated signaling pathway.

In the Response to Temperature Stimulus module, A0A1S4A548, annotated as a stromal 70-kDa heat shock-related protein, chloroplastic-like, and A0A1S3ZX95, annotated as a Heat shock factor-binding protein 1-like, showed relatively high connectivity in the PPI network (**Figure 6A**). A0A1S3ZX95 is annotated with transcription corepressor activity and may function as a transcriptional regulator of heat stress–responsive gene expression, whereas A0A1S4A548 is likely associated with protein folding and stress-related proteostasis. These genes may therefore represent candidate regulators or response factors linking regional environmental cues with transcriptional remodeling during leaf development.

**Figure 6:**
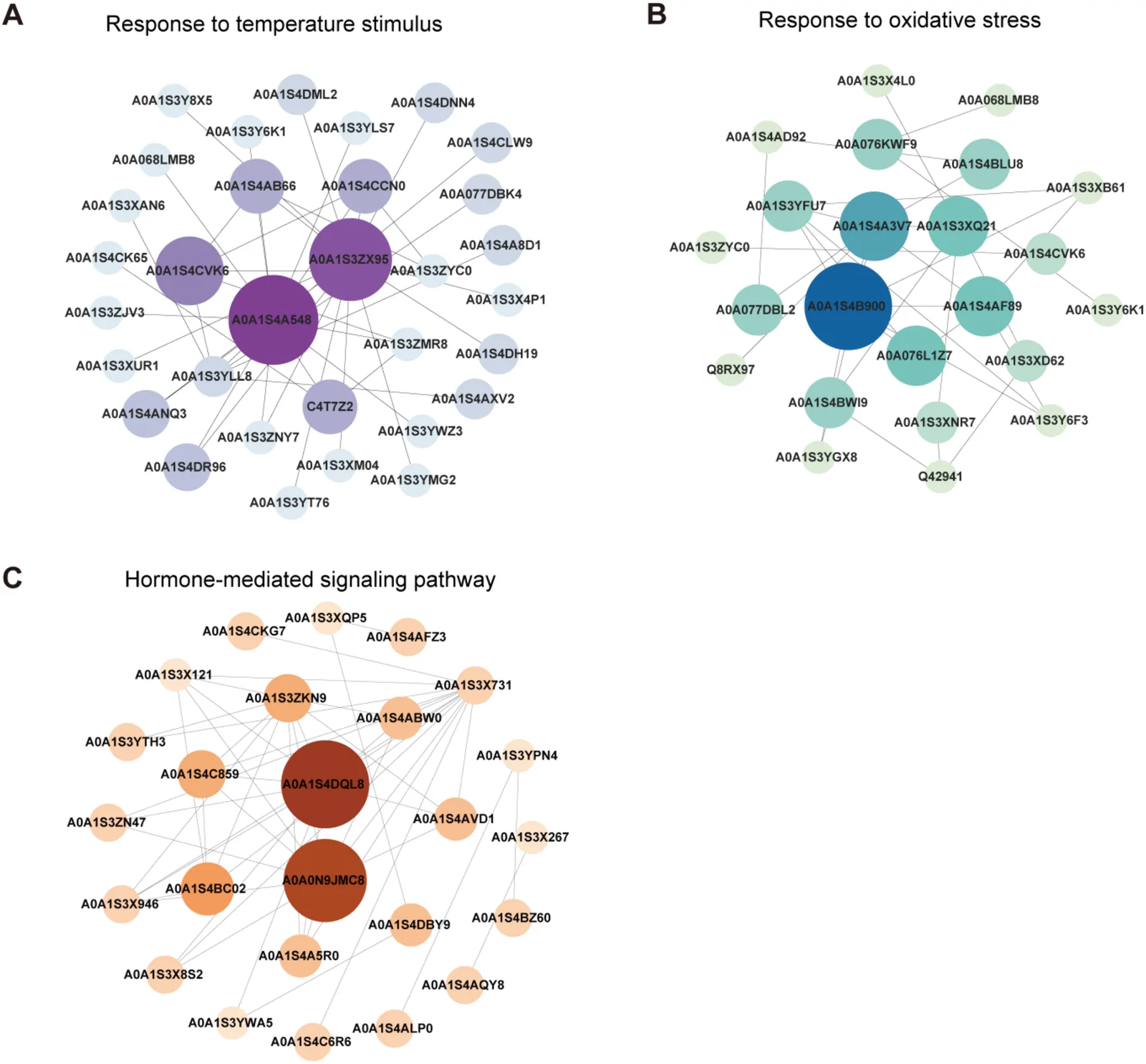
Protein–protein interaction networks of candidate environmental response modules. **(A)** PPI network of genes associated with the response to temperature stimulus. **(B)** PPI network of genes associated with the response to oxidative stress. **(C)** PPI network of genes associated with the hormone-mediated signaling pathway. In this layout, node size is proportional to the indegree (number of incoming interactions), highlighting key regulatory proteins within each network.

The oxidative stress-related module was also prominent in the representative strong-aroma comparison. Within this module, A0A1S4B900, annotated as Peroxiredoxin Q, chloroplastic, and A0A1S4A3V7, annotated as thioredoxin-dependent peroxiredoxin, were among the connected hub genes (**Figure 6B**). These proteins are associated with redox homeostasis and reactive oxygen species detoxification, suggesting that oxidative stress responses may contribute to the environmental sensitivity of aroma-related transcriptional programs. Given the known involvement of redox regulation in plant stress adaptation and secondary metabolism, these genes provide useful candidates for future functional validation.

In the hormone-mediated signaling module, several auxin-related genes showed reduced expression in the strong-aroma comparison, including A0A1S4DQL8 and A0A0N9JMC8, both annotated as auxin efflux carrier components, and A0A1S4BC02, annotated as an auxin responsive protein (**Figure 6C**). These observations suggest that auxin signaling may be linked to developmental and environmental transcriptional changes in tobacco leaves. However, because the present study is based on transcriptomic and network analysis, the relationship between auxin-related pathways and aroma formation remains hypothetical. Further experimental validation will be required to determine whether these candidate hub genes directly regulate aroma-related metabolic pathways or reflect broader changes in leaf maturation, stress responses, and resource allocation.

## Discussion

Tobacco aroma is a complex quality trait shaped by the interaction of genotype, developmental progression, environmental conditions, and post-harvest processing. In this study, transcriptome profiling across ten cultivars, three developmental stages, and three growing regions revealed that regional environment was the dominant source of global transcriptomic variation, followed by developmental stage, whereas cultivar identity contributed less to overall expression separation. Because aroma compounds were not directly quantified in this study, our findings should be interpreted as transcriptome-level associations with aroma-related biological processes rather than direct biochemical evidence of aroma formation. Nevertheless, the results provide a useful framework for understanding how ecological context and leaf maturation may modulate gene expression programs linked to tobacco quality.

The strong regional effect observed in the PCA analysis suggests that tobacco leaf transcriptomes are highly responsive to environmental context. Regional differences may reflect combined effects of climate, soil properties, cultivation conditions, and microbial communities, all of which can influence plant metabolism and stress responses (Dong and Lin, 2021; Bailey-Serres et al., 2019; Scheres and van der Putten, 2017; Zhang et al., 2022). In plants, environmental signals are often integrated through changes in photosynthesis, carbon allocation, hormone signaling, redox balance, and secondary metabolism. These processes are directly relevant to tobacco quality because carbohydrates, carotenoid-derived volatiles, phenylpropanoid-related compounds, terpenoids, and other specialized metabolites contribute to aroma precursor formation and sensory properties (Banozic et al., 2020; Popova et al., 2019). Thus, the regional separation of transcriptomic profiles likely reflects environment-dependent regulation of metabolic and stress-responsive pathways that can influence aroma-related potential.

Developmental progression was another major factor shaping the tobacco leaf transcriptome. Samples from S1 and S2 were transcriptionally similar, whereas S3 showed clear separation, indicating a major shift during late leaf maturation. This pattern is consistent with previous metabolomic and proteomic studies showing that tobacco leaf maturation is accompanied by extensive changes in proteins, fatty acids, alkaloids, and aroma precursors (Zhang et al., 2018; Chen et al., 2019). Our differential expression and Mfuzz analysis further showed that S3 was characterized by coordinated transcriptional remodeling, including downregulation of photosynthesis- and chloroplast-related pathways and upregulation of pathways associated with amino acid metabolism, lipid metabolism, cell wall and membrane biosynthesis, glutathione metabolism, and secondary metabolite biosynthesis. These changes are consistent with the transition from active photosynthetic growth toward mature leaf physiology, during which carbon resources may be redirected from primary photosynthetic activity toward structural remodeling, stress adaptation, and specialized metabolism.

The broad repression of photosynthesis-related genes is particularly relevant to aroma-associated metabolism. During leaf maturation and senescence, reduced photosynthetic activity and chloroplast remodeling are accompanied by chlorophyll degradation, altered carbon metabolism, and changes in the availability of metabolic precursors (He et al., 2022). In tobacco, carbohydrates are important contributors to quality and flavor development, and carotenoid metabolism provides precursors for carotenoid-derived volatile compounds (Banozic et al., 2020; Popova et al., 2019). Therefore, the observed decrease in photosynthesis, carbon fixation, and antenna protein pathways may reflect a developmental reallocation of metabolic flux that is associated with aroma precursor accumulation. At the same time, the enrichment of amino acid, lipid, and glutathione-related pathways suggests that nitrogen metabolism, membrane remodeling, and redox regulation may also participate in the maturation-associated biochemical environment that supports tobacco quality formation.

Our analysis further suggested that cultivars with different aroma characteristics may differ in transcriptional variability. Strong-aroma cultivars such as K326 and Yueyan96 showed higher gene expression CVs, whereas the light-aroma cultivar Hongda showed lower transcriptomic variability. Gene expression variation can provide phenotypic flexibility under changing environmental conditions and may contribute to metabolic diversity (Ferea et al., 1999; Oleksiak et al., 2005; Marais et al., 2013; Altschuler and Wu, 2010). In this context, higher transcriptomic variability in strong-aroma cultivars may indicate greater transcriptional plasticity and environmental responsiveness. This plasticity could facilitate stronger remodeling of stress, redox, and secondary metabolic pathways under different growing conditions. However, because aroma compounds were not directly measured, this relationship should be viewed as a hypothesis that requires future integration with metabolomic and sensory data.

The PPI and functional module analysis highlighted several candidate regulatory processes that may connect environmental responsiveness with aroma-related transcriptional remodeling. Temperature-response modules contained candidate hub genes annotated as heat stress transcription factors and heat shock-related proteins, suggesting that protein homeostasis and heat-responsive transcriptional regulation may contribute to regional adaptation of tobacco leaves. Oxidative stress modules included genes annotated as peptide-methionine sulfoxide reductase and thioredoxin-dependent peroxiredoxin, both of which are associated with redox homeostasis and reactive oxygen species detoxification. Redox regulation is closely linked to plant stress responses and secondary metabolism, and may influence the biochemical environment in which aroma precursors accumulate. In addition, auxin-related genes in the hormone-mediated signaling module showed reduced expression in the strong-aroma comparison. Auxin signaling is known to regulate growth, developmental timing, and resource allocation, suggesting that altered hormone signaling may interact with maturation and stress-response programs during tobacco leaf development. These candidate hub genes provide starting points for future functional validation, but their direct roles in aroma formation remain to be tested experimentally.

Together, our study suggests that tobacco aroma-related transcriptional programs are shaped by the combined effects of regional environment and developmental maturation. A general decline in photosynthesis-related pathways, together with activation of metabolic, redox, stress-responsive, and hormone-related processes, may reflect a coordinated transition from growth-oriented leaf physiology to mature leaf metabolic remodeling. By integrating large-scale transcriptomic profiling across cultivars, regions, and developmental stages, this work identifies candidate pathways and hub genes potentially involved in environmentally responsive aroma-related metabolism. Future studies combining transcriptomics with targeted metabolomics, volatile profiling, environmental measurements, and genetic or physiological validation will be essential to determine how these candidate regulatory modules contribute to tobacco aroma formation.

## Conclusions

This study provides a transcriptome-level framework for understanding how regional environment and developmental progression shape aroma-related gene expression programs in tobacco leaves. By profiling ten *Nicotiana tabacum* cultivars across three developmental stages and three growing regions, we found that growing region was the dominant source of global transcriptomic variation, followed by developmental stage, whereas cultivar identity had a weaker overall effect. Leaf maturation, particularly the transition from S1 to S3, was associated with broad downregulation of photosynthesis-, chloroplast-, and carbon fixation-related pathways and activation of metabolic, stress-responsive, redox-related, and secondary metabolic processes. We also found that strong-aroma cultivars showed higher transcriptomic variability, suggesting greater transcriptional plasticity and environmental responsiveness. Functional enrichment and PPI analysis further identified candidate modules and hub genes related to temperature response, oxidative stress, redox homeostasis, and hormone-mediated signaling. Although aroma compounds were not directly quantified, these findings highlight candidate pathways and regulatory genes potentially involved in environmentally responsive aroma-related metabolic remodeling, providing a basis for future functional validation, metabolomic integration, and cultivation or breeding strategies aimed at improving tobacco leaf quality.

## Data Availability

The datasets presented in this study can be found in online repositories. The names of the repository/repositories and accession number(s) can be found in the article/**Supplementary Material**.

## Supplementary material

Supplementary Table 1 Number of samples collected from each cultivar across three regions.

Supplementary Table 2 Gene information for each time-series expression cluster identified by Mfuzz analysis.

Supplementary Figure 1 Stratified PCA analysis of tobacco leaf transcriptomes under different grouping conditions. **(A)** PCA analysis of normalized gene expression values grouped by growing region within each developmental stage. Samples are colored by region: Chenzhou (CZ), Longshan (LS), and Yuxi (YX)**. (B)** PCA analysis grouped by developmental stage within each growing region. Samples are colored by developmental stage: central flowering period (S1), mature stage of the peduncle (S2), and middle leaf maturity stage (S3). **(C)** PCA analysis grouped by cultivar within each region or developmental-stage subset. Samples are colored by cultivar identity. Ellipses indicate the 95% confidence intervals for each group. These stratified analyses further evaluate the relative effects of growing region, developmental stage, and cultivar on global transcriptomic variation.

Supplementary Figure 2 Time-series clustering of developmental gene expression patterns across cultivars in Chenzhou. Mfuzz soft clustering was performed using gene expression profiles across three developmental stages: S1, central flowering period; S2, mature stage of the peduncle; and S3, middle leaf maturity stage. Clustering patterns are shown for each cultivar-region combination. Each panel contains six expression clusters, representing distinct temporal expression trajectories during tobacco leaf development. The y-axis shows normalized expression change, and the x-axis indicates developmental stage.

Supplementary Figure 3 Time-series clustering of developmental gene expression patterns across cultivars in Longshan. Mfuzz soft clustering was performed using gene expression profiles across three developmental stages: S1, central flowering period; S2, mature stage of the peduncle; and S3, middle leaf maturity stage. Clustering patterns are shown for each cultivar-region combination. Each panel contains six expression clusters, representing distinct temporal expression trajectories during tobacco leaf development. The y-axis shows normalized expression change, and the x-axis indicates developmental stage.

Supplementary Figure 4 Time-series clustering of developmental gene expression patterns across cultivars in Yuxi. Mfuzz soft clustering was performed using gene expression profiles across three developmental stages: S1, central flowering period; S2, mature stage of the peduncle; and S3, middle leaf maturity stage. Clustering patterns are shown for each cultivar-region combination. Each panel contains six expression clusters, representing distinct temporal expression trajectories during tobacco leaf development. The y-axis shows normalized expression change, and the x-axis indicates developmental stage.

Supplementary Figure 5 GO enrichment analysis of upregulated and downregulated developmental genes across regions. GO enrichment analysis of shared developmental gene sets in Chenzhou (CZ), Longshan (LS), and Yuxi (YX). Results are shown separately for biological process (GO_BP), molecular function (GO_MF), and cellular component (GO_CC). Dot size represents the gene ratio, and dot color indicates the adjusted P value. The x-axis shows region-specific upregulated and downregulated gene sets, and the y-axis shows enriched GO terms.

Supplementary Figure 6 Differentially expressed genes between S3 and S1 across cultivars and growing regions. **(A)** DEGs between S3 and S1 in Chenzhou. **(B)** DEGs between S3 and S1 in Longshan. **(C)** DEGs between S3 and S1 in Yuxi. Volcano plots showing differentially expressed genes (DEGs) identified by comparing the middle leaf maturity stage (S3) with the central flowering period (S1) for each cultivar within each growing region. Each subplot represents one tobacco cultivar. Red and blue points indicate significantly upregulated and downregulated genes, respectively, based on FDR < 0.01 and |log2 fold change> 1. Gray points indicate genes that did not meet the significance threshold. The numbers shown in each subplot indicate the counts of upregulated and downregulated DEGs.
