# Supplementary material for: Transcriptional signatures associated with environmental and developmental variation in tobacco aroma-related pathways across cultivars and regions
Source: Front Plant Sci. 2026 Oct 21;17:1854430. doi: 10.3389/fpls.2026.1854430 (PMC13731114; doi:10.3389/fpls.2026.1854430)
Supplement: Supplementary file 1 [file DataSheet1.docx]

**Supplementary Materials**

**Supplementary Table 1. Number of samples collected from each cultivar across three regions.**

| **Region** | **Chenzhou** | | | **Longshan** | | | **Yuxi** | | | **Total** |
| --- | --- | --- | --- | --- | --- | --- | --- | --- | --- | --- |
| Stage | S1 | S2 | S3 | S1 | S2 | S3 | S1 | S2 | S3 |  |
| Yunyan2 | 4 | 0 | 4 | 4 | 4 | 4 | 4 | 4 | 8 | 36 |
| K326 | 4 | 0 | 4 | 4 | 5 | 4 | 4 | 4 | 8 | 37 |
| G30 | 4 | 4 | 4 | 4 | 4 | 4 | 4 | 4 | 8 | 40 |
| Yueyan96 | 4 | 4 | 4 | 4 | 4 | 4 | 4 | 4 | 8 | 40 |
| Yunyan85 | 4 | 4 | 3 | 4 | 4 | 4 | 4 | 4 | 9 | 40 |
| Yunyan87 | 4 | 4 | 3 | 4 | 4 | 4 | 4 | 4 | 8 | 39 |
| Zhongyan103 | 4 | 0 | 4 | 4 | 4 | 4 | 4 | 4 | 8 | 36 |
| Zhongyan104 | 4 | 0 | 4 | 4 | 4 | 4 | 4 | 4 | 8 | 36 |
| Nanjiang3 | 4 | 4 | 4 | 4 | 4 | 4 | 4 | 4 | 8 | 40 |
| Hongda | 4 | 0 | 4 | 4 | 4 | 4 | 4 | 4 | 8 | 36 |
| Total | 40 | 20 | 38 | 40 | 41 | 40 | 40 | 40 | 81 | 380 |

**Supplementary Table 2.** **Gene information for each time-series expression cluster identified by Mfuzz analysis.**

See Excel.

**Supplementary figures**

**Figure S1. Stratified PCA analysis of tobacco leaf transcriptomes under different grouping conditions.**

**(A)** PCA analysis of normalized gene expression values grouped by growing region within each developmental stage. Samples are colored by region: Chenzhou (CZ), Longshan (LS), and Yuxi (YX)**.**

**(B)** PCA analysis grouped by developmental stage within each growing region. Samples are colored by developmental stage: central flowering period (S1), mature stage of the peduncle (S2), and middle leaf maturity stage (S3).

**(C)** PCA analysis grouped by cultivar within each region or developmental-stage subset. Samples are colored by cultivar identity. Ellipses indicate the 95% confidence intervals for each group. These stratified analyses further evaluate the relative effects of growing region, developmental stage, and cultivar on global transcriptomic variation.

**Supplementary Figure 2. Time-series clustering of developmental gene expression patterns across cultivars in Chenzhou.**
Mfuzz soft clustering was performed using gene expression profiles across three developmental stages: S1, central flowering period; S2, mature stage of the peduncle; and S3, middle leaf maturity stage. Clustering patterns are shown for each cultivar-region combination. Each panel contains six expression clusters, representing distinct temporal expression trajectories during tobacco leaf development. The y-axis shows normalized expression change, and the x-axis indicates developmental stage.

**Supplementary Figure 3. Time-series clustering of developmental gene expression patterns across cultivars in Longshan.**
Mfuzz soft clustering was performed using gene expression profiles across three developmental stages: S1, central flowering period; S2, mature stage of the peduncle; and S3, middle leaf maturity stage. Clustering patterns are shown for each cultivar-region combination. Each panel contains six expression clusters, representing distinct temporal expression trajectories during tobacco leaf development. The y-axis shows normalized expression change, and the x-axis indicates developmental stage.

**Supplementary Figure 4. Time-series clustering of developmental gene expression patterns across cultivars in Yuxi.**
Mfuzz soft clustering was performed using gene expression profiles across three developmental stages: S1, central flowering period; S2, mature stage of the peduncle; and S3, middle leaf maturity stage. Clustering patterns are shown for each cultivar-region combination. Each panel contains six expression clusters, representing distinct temporal expression trajectories during tobacco leaf development. The y-axis shows normalized expression change, and the x-axis indicates developmental stage.

**Supplementary Figure S5. GO enrichment analysis of upregulated and downregulated developmental genes across regions.**
GO enrichment analysis of shared developmental gene sets in Chenzhou (CZ), Longshan (LS), and Yuxi (YX). Results are shown separately for biological process (GO_BP), molecular function (GO_MF), and cellular component (GO_CC). Dot size represents the gene ratio, and dot color indicates the adjusted P value. The x-axis shows region-specific upregulated and downregulated gene sets, and the y-axis shows enriched GO terms.

**Supplementary Figure S6. Differentially expressed genes between S3 and S1 across cultivars and growing regions.** (**A**) DEGs between S3 and S1 in Chenzhou. (**B**) DEGs between S3 and S1 in Longshan. (**C**) DEGs between S3 and S1 in Yuxi. Volcano plots showing differentially expressed genes (DEGs) identified by comparing the middle leaf maturity stage (S3) with the central flowering period (S1) for each cultivar within each growing region. Each subplot represents one tobacco cultivar. Red and blue points indicate significantly upregulated and downregulated genes, respectively, based on FDR < 0.01 and |log2 fold change| > 1. Gray points indicate genes that did not meet the significance threshold. The numbers shown in each subplot indicate the counts of upregulated and downregulated DEGs.
